# The effectiveness of mHealth interventions for maternal, newborn and child health in low– and middle–income countries: Protocol for a systematic review and meta–analysis

**DOI:** 10.7189/jogh.04.010407

**Published:** 2014-06

**Authors:** Ulugbek B. Nurmatov, Siew H. Lee, Bright I. Nwaru, Mome Mukherjee, Liz Grant, Claudia Pagliari

**Affiliations:** 1 eHealth Research Group, Centre for Population Health Sciences, The University of Edinburgh Medical School, Edinburgh, Scotland, UK; 2 Health Services Research Unit, University of Edinburgh, Edinburgh, Scotland, UK; 3 Edinburgh Global Health Academy, The University of Edinburgh Medical School, Edinburgh, Scotland, UK

## Abstract

**Introduction:**

Rates of maternal, newborn and child (MNCH) mortality and morbidity are vastly greater in low– than in high–income countries and represent a major source of global health inequity. A host of systemic, economic, geopolitical and sociocultural factors have been implicated. Mobile information and communication technologies hold potential to ameliorate several of these challenges by supporting coordinated and evidence–based care, facilitating community based health services and enabling citizens to access health information and support. mHealth has attracted considerable attention as a means of supporting maternal, newborn and child health in developing countries and research to assess the impacts of mHealth interventions is increasing. While a number of expert reviews have attempted to summarise this literature, there remains a need for a fully systematic review employing gold standard methods of evidence capture, critical appraisal and meta–analysis, in order to comprehensively map, quality assess and synthesise this body of knowledge.

**Objectives:**

To undertake a systematic review and meta–analysis of studies evaluating the impacts of mobile technology–enabled interventions designed to support maternal, newborn and child health in low– and middle–income countries.

**Methods:**

16 online international electronic databases of published scientific abstracts and citations will be interrogated for the period 1990 to 2014 (no language restrictions) in order to identify relevant studies. Ongoing/unpublished studies will be identified through searching international trial repositories and consulting experts in the field. Study quality will be assessed using appropriate critical appraisal tools; including the Cochrane Handbook’s 7 evaluation domains for randomised and clinical trials, the Cochrane Effective Practice and Organisation of Care (EPOC) guidelines for other comparative study types, and the Effective Public Health Practice Project (EPHPP) quality assessment tools for observational studies. Blinded assessment by at least two reviewers, with arbitration by a third if necessary, will ensure rigour. Meta–analysis will be undertaken, where possible, using a random–effects model. Sensitivity and subgroup analyses will be reported. Publication bias will be assessed.

**Ethics and dissemination:**

Ethical approval is not required.

**Results:**

These will be presented in one manuscript. The review protocol is registered with the International Prospective Register for Systematic Reviews (PROSPERO) CRD42014008939.

Nowhere are global inequalities more starkly evident than in maternal, newborn and child health. For every 100 000 births in low income countries around 240 women die, compared with only 16 in high income countries, while a child is approximately 18 times more likely to die before the age of five years [1]. Although considerable progress has been made in meeting Millennium Development Goals 4 and 5 there remains a substantial gap between these aspirations and reality [2].

Preventable birth complications such as haemorrhage, obstructed labour and infection, which are exacerbated by poor pre– and post–natal care, account for a substantial proportion of these deaths [3,4]. These complications can also result in significant long–term health consequences for mothers, such as obstetric fistula, uterine prolapse, incontinence, depression, chronic infections and infertility, as well as mental or physical disability in their offspring [5,6].

While pathogens clearly play an important role, this excessive mortality and morbidity has been attributed largely to endemic failures in health and social care systems in low and middle income countries (LMIC) [3], set against a context of societal challenges such as lack of education and knowledge, delays in help seeking and poor nutrition. These factors are often exacerbated by gender discrimination, ethnic and religious division, and a lack of a social and political voice, as well as the more obvious economic and demographic barriers [2,7,8].

The problems described above have been compounded by historically weak information systems and poorly integrated infrastructures in LMIC [9]. However, the increasing penetration of mobile networks into these regions is opening up new opportunities to enable coordinated, accessible, safe, effective and citizen–centric health care. mHealth, or mobile health, refers to the use of wireless, portable information and communication technologies (ICT) to support health and health care [10]. The concept of mHealth remains somewhat poorly defined in the literature, although useful taxonomies are beginning to emerge [11]. It may be thought of as involving various *devices* such as cell– and smart–phones, personal digital assistants, tablet computers, laptops and digital point–of–care testing devices, *delivery modes* such as voice, text, images, or video, and *applications* such as public health messaging, personalised behaviour change interventions, workflow management, health surveillance, access to patient records, clinical decision support, education, diagnostics and remote care provision. Thus mHealth solutions may be configured to support patients, professionals and health systems. Although mHealth has attributes in common with ‘telemedicine’ and ‘telehealth’ (enabling care at a distance) and other areas of ‘health informatics’ (eg, via digital records) the term is reserved exclusively for mobile and wireless digital tools and interventions.

Although the area of mHealth is relatively young, it has attracted considerable attention from, and investment by, donors, the private sector and development agencies as a means of alleviating a range of global health challenges. Among them, maternal, newborn and child health are key priorities, along with the monitoring and management of HIV and other infectious diseases. International collaborations such as the mHealth Alliance and development agencies like USAID are increasingly documenting experiences of mHealth implementation projects in this area, one of the best known being MAMA (Mobile Alliance for Maternal Action), a patient messaging system that has been implemented in several LMIC [12,13].

While the significance of mHealth is understood, evidence of its potential value and impact on maternal, newborn and child health is less clear. Intermediate outcomes, such as improved antenatal attendance through the use of SMS appointment reminders [14], are increasingly being reported, although evidence of impacts on maternal and child mortality and morbidity rates is rarer [15]. Significantly, the recent World Health Organisation guideline on postnatal care of the mother and newborn identifies a need to evaluate the potential role of mHealth in improving patient outcomes [16].

To date, the majority of mHealth implementation projects in LMIC have tended to be small–scale, donor–funded initiatives, which have taken place without the benefit of an adequate evidence–base, and have not themselves been configured with research in mind. However the area is beginning to attract greater research attention and funding, with a growing body of studies examining the appropriate design of mHealth interventions for patients and professionals, their impacts on the processes and outcomes of care, and the barriers and facilitators to scaling up [14,15]. Challenges include the difficulties of undertaking rigorous trials in projects driven by development or policy goals, attributing cause and effect where both interventions and the environments in which they are delivered are complex, and designing and targeting interventions for greatest impact. For example, a recent trial of text–messaging to encourage attendance at antenatal care suggested improved uptake of preventive care services but the authors acknowledge that randomisation at the level of health facilities rather than individual patients may have failed to capture women at earlier stages of pregnancy, for whom community–based recruitment might have been more appropriate [11]. Reviewers such as Tomlinson et al have also stressed the importance of adapting tools to suit the context and culture of care in order to optimise their likely impacts [15].

A number of systematic reviews on the topic of mHealth exist but these are not ideally suited to establishing impacts on MCNH outcomes in LMIC, in some cases due to the absence of certain databases likely to capture research from these regions [17,18] or because they are concerned more with methodological and process issues than patient outcomes [19]. The majority of secondary literature specific to mHealth for MCNH in LMIC consists of broad expert reviews summarising activities and aspirations and descriptive case studies illustrating how mobile technologies are being used to support the strengthening of health systems [20-22]. Although these provide valuable insights, many are vision–driven rather than evidence–seeking and the influence of the mobile telecommunications industry can be hard to disentangle. A project to map the state of the evidence on mHealth and MCNH was recently undertaken by Philbrick for the mHealth Alliance [22]. This converged a literature review with a landscape scan of existing projects and interviews with key respondents to provide a breakdown of studies in this area, their limitations and their implications for research and policy. While the report is useful, the mixed–methods ‘gap analysis’ exercise was not configured as a formal systematic review and is thus likely to have missed important sources of evidence and steps in the critical appraisal of study methodology, which would be expected in a classic systematic review. Two other quasi–systematic literature reviews addressed the topic using basic search terms and a subset of six databases [23,24]. A systematic review project selected for funding by the Alliance for Health Policy and Systems Research in 2011 [25] remains unpublished, as is the case for a systematic review undertaken for a Master’s degree project at the University of Edinburgh, which focused only on SMS messaging for prenatal care [26]. We are therefore confident that there is a clear need for a new systematic review employing gold standard methods of evidence capture, critical appraisal and meta–analysis, in order to comprehensively map, quality assess and synthesize existing evidence of the impact of mobile technology–enabled interventions on maternal, newborn and child health in low and middle income countries. The current study will follow the rigorous methods advocated by the International Cochrane Collaboration in order to achieve these aims and the findings will be interpreted with reference to relevant theoretical and policy perspectives, in order to derive recommendations for research and practice. In this protocol we describe the steps that are planned in order to undertake this systematic review.

## DESIGN

This is the protocol for a systematic review and meta–analysis of the literature.

## AIMS AND OBJECTIVES

To undertake a systematic review and meta–analysis of studies evaluating the impacts of mobile technology–enabled interventions designed to support maternal, newborn and child health in low– and middle–income countries.

## INCLUSION AND EXCLUSION CRITERIA

### Included interventions

We are interested in any intervention delivered using mobile ICT, which is designed to support the health of pregnant women and their unborn children, women during and after childbirth, newborns, infants and children up to five years and the national, state, city, or community level in an LMIC setting.

Mobile ICT refers to portable, wireless digital *devices* usually (although not exclusively) supported by networked mobile or satellite communications infrastructures, such as cell–phones, smart–phones, satellite phones, personal digital assistants, enterprise digital assistants, tablet computers, laptops, portable media players and gaming consoles, Radio Frequency Identification Device (RFID) tags, Global Positioning System (GPS) trackers and digital diagnostic devices [27]. mHealth interventions involve a range of *delivery modes* such as voice calling, Voice over Internet Protocol (VoIP), text messaging via Short Message Service (SMS), transfer of still or moving images via Multimedia Message Service (MMS), multimedia downloads, or live video [17]. Within the scope of this review we include all *applications* of these technologies for directly supporting MCNH *patients* – such as public health messaging, personalised behaviour change communications, self–care information and remote care provision, as well as interventions designed to enable trained or lay *health workers* to provide better care to patients – such as electronic medical records or care plans for supporting individualised care; decision support tools for informing screening or intervention decisions, workflow planning applications, clinical documentation tools, global positioning tools for patient tracking and portable point–of–care testing devices able to transmit data via mobile phone or satellite networks.

### Included study types

The following study designs will be potentially eligible for inclusion:

• Randomised controlled trials (RCTs, quasi–RCTs, Controlled Clinical Trials – CCTs). Study designs included by The Cochrane Effectiveness Practice and Organisational Care (EPOC) group – (controlled before–and–after studies, interrupted time series studies),

• Cohort and case–control studies.

### Types of participants included

The following types of participant will be potentially eligible for inclusion:

• Pregnant women,

• Women in ante–natal, intra–natal and postnatal periods,

• Newborns,

• Children aged 0–5 years,

• Health workers through which an intervention aimed at improving the health of the above groups is mediated.

### Excluded interventions

This review will exclude interventions focused on reproductive health (eg, promotion of HPV vaccination), sexual health (eg, domestic violence reporting) or sexually transmitted diseases (eg, antiretroviral treatment (ART) compliance reminders), unless pregnant women or mothers of newborns or children 0–5 years are specifically targeted (many pregnant women in LMIC have HIV).

mHealth interventions aimed at managerial or financial aspects of health systems (such as stock control or accounting) will not be included in this review.

Studies describing physically ‘mobile’ clinics or services will be excluded unless mobile ICT is a fundamental medium through which the service is delivered.

### Excluded study types

The following study types will be excluded:

• Studies undertaken in high–income countries,

• Expert opinion,

• Descriptive case studies and case series,

• Technical reports and reviews.

### Types of participants excluded

The following types of participants will be excluded:

• Men, adolescent males and boys over the age of 5 years,

• Women, adolescent females and girls over the age of 5 years who are not pregnant, have not recently given birth or are not caring for their child aged 0–5,

• Facility managers and government decision makers not directly involved in the care of patients. (mHealth interventions such as clinical dashboards may support higher–level administrative functions associated with the operation of MCNH services but do so indirectly).

### Types of comparisons

Included studies will be those comparing the mHealth intervention with usual care, another intervention or a non–exposed control group.

### Types of outcome measures

The following outcome measures will be included:

• Primary outcomes: all outcome measures indicative of maternal mortality; maternal morbidity; newborn and child mortality; newborn and child morbidity,

• Secondary outcomes: number of planned antenatal and post natal visits; number of unscheduled care visits and emergency care incidents; quality of life; quality of care (delivery by skilled birth attendants, appropriate use of evidence–based medical and obstetric interventions where available); self–efficacy; cost–effectiveness; immunisation cover; child developmental milestones and mHealth intervention–related adverse events.

## SEARCH METHODS

Eligible study reports will be identified from the following sources:

• The Cochrane Library (Cochrane Database of Systematic Reviews, Cochrane Central Register of Controlled Trials (CENTRAL), Cochrane Methodology Register), MEDLINE, EMBASE, CINAHL, PsycINFO, AMED, Global Health, TRIP, ISI Web of Science (Science and Social Science Index), WHO Global Health Library, IndMed, PakMediNet, KoreaMed, NHS Health Technology Assessment Database, African Index Medicus (encompassed in the WHO Global Health Library), and POPLINE. Studies will be identified using subject headings appropriate to each database as well as free–text terms. In addition, reference lists of articles of interest and citations to included articles will be screened for additional eligible published studies.

• Unpublished and in progress studies will be identified from the following trial registries: www.clinicaltrials.gov; www.controlled–trials.com; www.anzctr.org.au; http://www.who.int/ictrp/en/.

• Expert consultation.

The search strategy is presented in detail in **Tables 1**** and ****2**.

**Table 1:** Search strategy: Medline format

1. (eHealth or e–Health).mp.
2. (mHealth or m–health or mobile health).mp.
3. Telemedicine/ or (telecare or telehealth care or mobile telehealth care or mobile telemedicine or mCare or m–care).mp.
4. apps or mobile applications/
5. (mobile communication or mobile technology or mobile devic*).mp.
6. Computers, Handheld/ or Microcomputers/ or (tablet computers or mobile tablet computers or mobile technolog*).mp.
7. Communication satellite.mp.
8. Cellular phone/ or (cellular phone* or cell phone or mobile phone).mp.
9. MP3 player*.mp.
10. Text Messaging/ or (texting or text messag* or messag* or text* or short message or SMS or multimedia technol* or multimedia messag* or multi–media messag*).mp.
11. (Personal digital assistant* or PDA).mp.
12. (Smartphone or smart–phone).mp.
13. (podcast* or pod–cast*).mp.
14. Social media/ or Social networking/ or (Twitter or Facebook).mp.
15. (Global positioning system or GPS).mp.
16. Radio Frequency Identification Device/ or RFID.mp.
17. or/1–16
18. Pregnancy/ or Pregnant women/ or Pregnancy outcome/
19. Parturition/ or childbirth.mp.
20. Obstetrics/
21. Maternal Health Services/ or matern*
22. (pregnan* or maternal or maternal health).mp.
23. Labor, or Labour or Obstetric/
24. Delivery, Obstetric/
25. Midwifery/ or Traditional Birth Attendant.mp.
26. Postpartum period/ or puerperium.mp.
27. Delayed delivery.mp. or three delays.mp.
28. Pregnancy complications/ or Obstetric Labor complications/ or Obstetric Labor, Premature/ or Puerperal Disorders/ or Depression, Postpartum/ or Maternal Mortality/
29. Infant, Newborn/ or neonat*.mp.
30. Prenatal Care/ or Perinatal Care/ or Postnatal Care.mp.
31. (Antenatal care or intrapartum care or postpartum care or post–partum care or puerperal care).mp.
32. (Perinatal complication* or postnatal complication*).mp.
33. Birth injuries/
34. Breastfeeding/ or (breastfeeding or breast–feeding).mp.
35. or/18–34
36. Intervention studies/ or experimental studies.mp.
37. analytical stud*.mp.
38. Clinical trial/ or Controlled Clinical Trial/ or Randomized Controlled Trial/ or (clinical trial or controlled clinical trial or randomized controlled trial).mp.
39. Double–Blind Method/ or Single–Blind Method/ or (double–blind design or single–blind design).mp.
40. Placebos/ or Random Allocation/ or random*.mp.
41. (Controlled before and after stud*).mp.
42. Interrupted time series.mp.
43. Cohort studies/ or (cohort stud* or cohort).mp.
44. (control or healthy control).mp.
45. Case–control studies/ or case–control stud*.mp.
46. or/36–45
47. 17 and 35 and 46
48. limit 47 to yr = ”1990–2014”
49. limit 48 to human

**Table 2:** Search strategy: free–field format

(mHealth or m–Health or eHealth or e–Health or telemedicine or mobile health or mobile telehealth care or mobile phone or cellular phone or personal digital assistant or mobile tablet computers or smart phone or mobile technology or apps or mobile applications or text messag* or short messag* or SMS or multimedia messag*)
AND
(child* or infant* or baby or babies or neonatal or newborn* or preterm* or prematur* or pregnan* or pregnant women or mother* or obstetric labor or obstetric labour or obstetric delivery or obstetric labor complications or midwifery or traditional birth attendant or perinatal care or prenatal care or antenatal care or intrapartum care or postnatal care or perinatal complications or postnatal complications)
AND
(analytical stud* or epidemiologic* or compar* or evaluat* or follow–up or follow up or prospective or retrospective observation* or cohort or case–control or trial* or RCT or controlled before and after study or interrupted time series or intervention* or prospective or retrospective or control* or double–blind or single–blind or random*)

Restrictions include:

• Time span: 1990–2014. (Rationale: the first mobile–health technology interventions started in the early 1990s) [20].

• Language: none (for foreign language papers translations will be sought)

• Countries identified as low or medium income according to the United Nations Human Development Report released in March 2013 [28]. We are aware that the position of countries in such indices changes over time and will note the date of the articles. We will also include articles referred to using the generic terms used by other authors to describe LMIC (eg, developing country, emerging economy) [29,30].

## STUDY SELECTION

Each author will be assigned one or more databases to search using an appropriately adapted version of the strategy described above (mindful of database differences). Retrieved titles and abstracts will be collated and distributed to pairs of authors for independent screening in order to identify potentially eligible studies. Disagreement will be resolved by consensus, or arbitration involving a third author where necessary. Full text articles will be retrieved for selected studies, and two authors will assess whether each of these meets the set inclusion criteria. Disagreement will be resolved by discussion amongst reviewers, with referral to a third author if necessary. Reasons for exclusion of studies will be noted. All authors will discuss and agree the refined list of included studies.

## QUALITY ASSESSMENT AND ANALYSIS

The assessment and documentation of the methodological quality of included controlled trials will follow the Cochrane approach using the methods detailed in section eight of the Cochrane Handbook for Systematic Reviews of Interventions [31]. Intervention studies will be assessed using the Cochrane Effectiveness and Practice Organisation of Care (EPOC) guidelines [32,33]. The following seven parameters will be used to assess trial quality: random sequence generation; allocation concealment; blinding of participants and personnel; blinding of outcome assessment; incomplete outcome data; selective reporting; and other biases. Each parameter of trial quality will be graded: A – low risk of bias; B – moderate risk of bias; C – high risk of bias and an overall assessment for each controlled trial using the same three criteria will be made. Observational studies will be similarly assessed using the Effective Public Health Practice Project (EPHPP) quality assessment tool for quantitative studies [34]. Reviewers will not be masked to study details. Agreement of reviewers on methodological quality assessment will be assessed and disagreements will be resolved by discussion.

All assessments of study quality will be performed by at least two reviewers (UN, CP) with any disagreement resolved by consensus, or arbitration via a separate reviewer where necessary.

## DATA EXTRACTION

Two reviewers will independently extract data using customised data extraction forms. The following information will be extracted:

• Author and year,

• National affiliation of author and funding source,

• Country in which the study took place,

• Study design,

• Healthcare setting,

• Target users,

• Type of mHealth intervention – device; delivery mode; application type; stated purpose of intervention; theoretical basis if specified,

• Range of outcome measures described – maternal mortality and morbidity, newborn and child mortality and morbidity, emergency care, quality of life, quality of care (delivery by skilled birth attendants, appropriate use of evidence–based medical and obstetric interventions where available), immunisation rates, and cost–effectiveness of interventions,

• Key findings from each included study will be summarised and tabulated.

## DATA ANALYSIS

Data will be presented in tabular and narrative form. Where possible, meta–analyses will be performed on methodologically comparable studies (comparable particularly with regards to the study design, type of ICT and endpoint measures studied and methods used to assess these) reporting main, primary, and secondary outcomes. The meta–analysis results will be presented in forest plots. The choice of statistical tests will depend on the nature of the outcome variable. Application of either a fixed effect or random effects model will be dependent on the degree of heterogeneity. Heterogeneity will be assessed both qualitatively and quantitatively using I^2^ statistic. Where possible, adjusted effect estimates will be pooled in meta–analyses using generic inverse–variance analysis. Point estimates and 95% confidence intervals will be reported for all analyses. Sensitivity analyses will be performed in subgroups of study quality and of design characteristics (eg, randomised vs non–randomised; prospective vs retrospective). Where relevant data are missing, we will contact authors to request these. In addition, will undertake appropriate sensitivity analyses to address the different scenarios of missingness to be observed by making appropriate assumptions of each missing scenario. Finally, we will provide relevant discussion of the influence of missing data on the observed findings.

Where the number of included studies per outcome is sufficient, publication bias will be assessed visually through Funnel plots and tested by Egger’s regression test [35] and Begg’s rank correlation test [36].

## ETHICS AND DISSEMINATION

### Ethical issues

As only previously published studies are included and reported in the review, no additional formal ethical assessment and no informed consent is required.

### Publication plan

The systematic review protocol is registered with the International Prospective Register of Systematic Reviews (PROSPERO) CRD42014008939 (http://www.crd.york.ac.uk/prospero). Findings will be summarised in a single manuscript.

### Timeline

Start date: 6 January 2014

Finishing date: 30 June 2014

Reporting date 30 June 2014

## References

[1] UNICEF. WHO, World Bank, UN-Desa Population Division. Levels and Trends in Child Mortality: Report 2013, Geneva: World Health Organisation, 2013. Available at: http://www.who.int/maternal_child_adolescent/documents/levels_trends_child_mortality_2013/en/index.html Accessed 3 January 2014.

[2] World Health Organisation. Children: reducing mortality. Fact sheet 178. 2012. Available at: http://www.who.int/mediacentre/factsheets/fs178/en/index.html Accessed: 3 January 2014.

[3] United Nations Children’s Fund. State of the world's children 2008: Maternal and newborn health. New York: UNICEF, 2008. Available at: http://www.unicef.org/publications/index_47127.html# Accessed: 3 January 2014.

[4] Black RE, Cousens S, Johnson HL, Lawn JE, Rudan I, Bassani DG (2010). Global, regional, and national causes of child mortality in 2008: a systematic analysis.. Lancet.

[5] Wulf SK, Johns N, Lozano R (2013). Non-fatal burden of maternal conditions: country-level results from the GBD 2010 Study.. Lancet.

[6] Mwaniki MK, Atieno M, Lawn JE, Newton CR (2012). Long-term neurodevelopmental outcomes after intrauterine and neonatal insults: a systematic review.. Lancet.

[7] Ronsmans C, Graham WJ (2006). Maternal mortality: who, when, where, and why.. Lancet.

[8] Van Malderen C, Van Oyen H, Speybroeck N (2013). Contributing determinants of overall and wealth-related inequality in under-5 mortality in 13 African countries.. J Epidemiol Community Health.

[9] Kerber KJ, de Graft-Johnson JE, Butta ZA, Okong P, Starrs A, Lawn JE (2007). Continuum of care for maternal, newborn, and child health: from slogans to service delivery.. Lancet.

[10] Istepanian RSH, Laxminarayan S, Pattichis CS, editors. M-Health: Emerging Mobile Health Systems. New York: Springer, 2005.

[11] Labrique AB, Vasudevan L, Kochi E, Fabricant R, Mehl G (2013). mHealth innovations as health systems strengthening tools: 12 common applications and a visual framework.. Glob Health Sci Pract..

[12] mHealth Alliance. New innovation grantees aim to reach 4.5 million people with mobile health services for improving reproductive, maternal and child health. Available at: http://mhealthalliance.org/media-a-resources/press-releases/156-new-innovation-grantees-aim-to-reach-45-million-people-with-mobile-health-services-for-improving-reproductive-maternal-and-child-health Accessed: 26 May 2014.

[13] Mendoza G, Okoko L, Morgan G, Konopka S. mHealth compendium. Volume Two. May 2013. Arlington, VA: African Strategies for Health Project, Management Sciences for Health. Available at: https://www.mhealthworkinggroup.org/sites/mhealthwg.org/files/usaid_mhealth_compendium_vol_2_-_final_0.pdf Accessed: 26 May 2014.

[14] Lund S, Nielsen BB, Hemed M, Boas IM, Said A, Said K (2014). Mobile phones improve antenatal care attendance in Zanzibar: a cluster randomised controlled trial.. BMC Pregnancy Childbirth.

[15] Tomlinson M, Rotheram-Borus MJ, Swartz L, Tsai AC (2013). Scaling up mHealth: Where is the evidence?. PLoS Med.

[16] WHO. WHO recommendations on Postnatal care of the mother and newborn. Report 2013, Geneva: World Health Organisation, 2013. Available at: http://apps.who.int/iris/bitstream/10665/97603/1/9789241506649_eng.pdf Accessed: 1 April 2014. 24624481

[17] Free C, Phillips G, Watson L, Galli L, Felix L, Edwards P (2013). The effectiveness of mobile-health technologies to improve health care service delivery processes: a systematic review and meta-analysis... PLoS Med.

[18] Free C, Phillips G, Watson L, Galli L, Felix L, Edwards P (2013). The effectiveness of mobile-health technology-based health behaviour change or disease management interventions for health care consumers: a systematic review.. PLoS Med.

[19] Chib A, van Velthoven MH, Car J (2014). mHealth adoption in low-resource environments: a review of the use of mobile healthcare in developing countries.. J Health Commun.

[20] Consulting VW. mHealth for Development: the opportunity of mobile technology for healthcare in the developing world. Washington, D.C. and Berkshire, UK: UN Foundation-Vodafone Foundation Partnership, 2009. Available at: http://www.globalproblems-globalsolutions-files.org/unf_website/assets/publications/technology/mhealth/mHealth_for_Development_full.pdf Accessed: 24 January 2014.

[21] Rotheram-Borus MJ, Tomlinson M, Swendeman D, Lee A, Jones E. Standardized functions for smartphone applications: Examples from maternal and child health. Int J Telemed Appl. 2012;2012:973237. 10.1155/2012/973237 PMC3530862 23304136

[22] Philbrick WC. mHealth and MNCH: State of the evidence. Trends, gaps, stakeholder needs, and opportunities for future research on the use of mobile technology to improve maternal, newborn, and child health. mHealth Alliance, UN Foundation Jan 2013. Available at: http://www.mhealthworkinggroup.org/sites/mhealthwg.org/files/philbrick_mnch_final.pdf Accessed: 3 January 2014.

[23] Noordam AC, Kuepper BM, Stekelenburg J, Milen A (2011). Improvement of maternal health services through the use of mobile phones.. Trop Med Int Health.

[24] Tamrat T, Kachnowski S (2012). Special delivery: An analysis of mhealth in maternal and newborn health programs and their outcomes around the world.. Matern Child Health J.

[25] Mutwiwa S. (PI) Effectiveness of mobile telephone services to improve maternal and neonatal health in low and middle income countries. Sponsored by the Alliance for Health Policy and Systems Research, and referred to in their newsletter dated July 2011. Available at: http://www.who.int/alliance-hpsr/alliancehpsr_newsletter21.pdf Accessed: 3 January 2014.

[26] Roofe M. Short message service only m-health interventions for prenatal care in developing countries: A systematic review. MSc Thesis. Edinburgh: University of Edinburgh Health Informatics Programme, 2012.

[27] Perry M, O’Hara K, Sellen A, Brown B, Harper R (2001). Dealing with mobility: understanding access anytime, anywhere.. ACM Trans Comput Hum Interact.

[28] United Nations Human Development Report 2013. The Rise of the South: Human Progress in a Diverse World. New York: United Nations Development Programme, 2013. Available at: http://hdr.undp.org/sites/default/files/reports/14/hdr2013_en_complete.pdf Accessed: 25 January 2014.

[29] Pande S, Hiller JE, Nkansah N, Bero L (2013). The effect of pharmacist-provided non-dispensing services on patient outcomes, health service utilisation and costs in low and middle-income countries.. Cochrane Database Syst Rev.

[30] Dudley L, Hviding K, Paulsen E (2009). The effectiveness of policies promoting facility-based deliveries in reducing maternal and infant morbidity and mortality in low and middle-income countries (Protocol).. Cochrane Database Syst Rev.

[31] Higgins JPT. Green S eds. Cochrane handbook for systematic reviews of interventions Version 5.1.0. The Cochrane Collaboration 2011. Available at: http://www.cochrane.org/training/cochrane-handbook Accessed: 29 January 2014.

[32] Cochrane Effective Practice and Organisation of Care Group. Available at: http://epoc.cochrane.org/ Accessed: 3 January 2014.

[33] Cochrane Effective Practice and Organisation of Care Review Group (EPOC). Data Collection Checklist. Available at: http://epoc.cochrane.org/sites/epoc.cochrane.org/files/uploads/datacollectionchecklist.pdf Accessed: 29 January 2014.

[34] Effective Public Health Practice Project (EPHPP). Quality assessment tool for quantitative studies. Available at: www.ephpp.ca Accessed: 20 December 2013.

[35] Egger M, Davey Smith G, Schneider M, Minder C (1997). Bias in meta-analysis detected by a simple, graphical test.. BMJ.

[36] Begg CB, Mazumdar M (1994). Operating characteristics of a rank correlation test for publication bias.. Biometrics.

